# Supplementary material for: Prevalence and determinants of care needs among older people in Ghana
Source: PLoS One. 2022 Feb 15;17(2):e0263965. doi: 10.1371/journal.pone.0263965 (PMC8846497; doi:10.1371/journal.pone.0263965)
Supplement: Attachment — Submitted filename: Reviewer #1 annotated manuscript.pdf [file pone.0263965.s004.pdf]

PLOS ONE

Prevalence and determinants of care needs among older people in Ghana

--Manuscript Draft--

**Manuscript Number:**	PONE-D-21-10419
**Article Type:**	Research Article
**Full Title:**	Prevalence and determinants of care needs among older people in Ghana
**Short Title:**	Prevalence and determinants of care needs among older people in Ghana
**Corresponding Author:**	Kwamina Abekah-Carter<br>University of Ghana<br>Accra, GHANA
**Keywords:**	Care needs; WHO-ICF; older people; Ghana
**Abstract:**	Given the increase in longevity among older people in Ghana, as well as the likely occurrence of functional disability in their later years, the understanding of the care needs of Ghanaian older people have become more critical. This cross-sectional study examined the prevalence and determinants of care needs among older people in Ghana. Data was gathered from a sample of 400 older people from the Komfo Anokye Teaching Hospital in Southern Ghana. Care needs were assessed by one question "Do you regularly need help with daily tasks because of long-term illness, disability or frailty?" Multivariate logistic regression was used to test the association between care needs and independent variables. The majority of the respondents (81%), particularly women (54%) reported a need for care in completing their daily tasks. The determinants of care needs were mainly functional disability (AOR=1.07 95%CI: 1.05-1.09, p<0.001) and the lack of government support (AOR=3.96 95%CI: 1.90-8.25, p<0.001). Functional disability and absence of government support are the major factors that need to be prioritized in addressing increased demand for care related to daily tasks among older people in Ghana.
**Order of Authors:**	Kofi Awuviry-Newton
	Kwadwo Ofori-Dua
	Charles Selorm Deku
	Kwamina Abekah-Carter
	Victoria Awortwe
	George Ofosu Oti
**Additional Information:**	
**Question**	**Response**
**Financial Disclosure**<br><br>Enter a financial disclosure statement that describes the sources of funding for the work included in this submission. Review the submission guidelines for detailed requirements. View published research articles from *PLOS ONE* for specific examples.<br><br>This statement is required for submission and **will appear in the published article** if the submission is accepted. Please make sure it is accurate.	No - Include this sentence at the end of your statement: The funders had no role in study design, data collection and analysis, decision to publish, or preparation of the manuscript.

Powered by Editorial Manager® and ProduXion Manager® from Aries Systems Corporation

**Unfunded studies**<br>Enter: *The author(s) received no specific funding for this work.*<br><br>**Funded studies**<br>Enter a statement with the following details:<br>• Initials of the authors who received each award<br>• Grant numbers awarded to each author<br>• The full name of each funder<br>• URL of each funder website<br>• Did the sponsors or funders play any role in the study design, data collection and analysis, decision to publish, or preparation of the manuscript?<br>• **NO** - Include this sentence at the end of your statement: *The funders had no role in study design, data collection and analysis, decision to publish, or preparation of the manuscript.*<br>• **YES** - Specify the role(s) played.<br><br>* typeset	
**Competing Interests**<br><br>Use the instructions below to enter a competing interest statement for this submission. On behalf of all authors, disclose any competing interests that could be perceived to bias this work—acknowledging all financial support and any other relevant financial or non-financial competing interests.<br><br>This statement is required for submission and **will appear in the published article** if the submission is accepted. Please make sure it is accurate and that any funding sources listed in your Funding Information later in the submission form are also declared in your Financial Disclosure statement.<br><br>View published research articles from *PLOS ONE* for specific examples.	The authors have declared that no competing interests exist.

**NO authors have competing interests**<br><br>Enter: *The authors have declared that no competing interests exist.*<br><br>**Authors with competing interests**<br><br>Enter competing interest details beginning with this statement:<br><br>*I have read the journal's policy and the authors of this manuscript have the following competing interests: [insert competing interests here]*<br><br>* typeset	
**Ethics Statement**<br><br>Enter an ethics statement for this submission. This statement is required if the study involved:<br><br>• Human participants<br>• Human specimens or tissue<br>• Vertebrate animals or cephalopods<br>• Vertebrate embryos or tissues<br>• Field research<br><br>Write "N/A" if the submission does not require an ethics statement.<br><br>General guidance is provided below. Consult the submission guidelines for detailed instructions. **Make sure that all information entered here is included in the Methods section of the manuscript.**	Ethical approval (H-2018-0163) for this analysis was obtained from The University of Newcastle in Australia. Informed consent was obtained from the director of Komfo Anokye Teaching Hospital and study participants.

Powered by Editorial Manager® and ProduXion Manager® from Aries Systems Corporation

**Format for specific study types**<br><br>**Human Subject Research (involving human participants and/or tissue)**<br>• Give the name of the institutional review board or ethics committee that approved the study<br>• Include the approval number and/or a statement indicating approval of this research<br>• Indicate the form of consent obtained (written/oral) or the reason that consent was not obtained (e.g. the data were analyzed anonymously)<br><br>**Animal Research (involving vertebrate animals, embryos or tissues)**<br>• Provide the name of the Institutional Animal Care and Use Committee (IACUC) or other relevant ethics board that reviewed the study protocol, and indicate whether they approved this research or granted a formal waiver of ethical approval<br>• Include an approval number if one was obtained<br>• If the study involved *non-human primates*, add *additional details* about animal welfare and steps taken to ameliorate suffering<br>• If anesthesia, euthanasia, or any kind of animal sacrifice is part of the study, include briefly which substances and/or methods were applied<br><br>**Field Research**<br><br>Include the following details if this study involves the collection of plant, animal, or other materials from a natural setting:<br>• Field permit number<br>• Name of the institution or relevant body that granted permission	
**Data Availability**<br><br>Authors are required to make all data underlying the findings described fully available, without restriction, and from the time of publication. PLOS allows rare exceptions to address legal and ethical concerns. See the PLOS Data Policy and FAQ for detailed information.	No - some restrictions will apply

A Data Availability Statement describing where the data can be found is required at submission. Your answers to this question constitute the Data Availability Statement and **will be published in the article**, if accepted.<br><br>**Important:** Stating 'data available on request from the author' is not sufficient. If your data are only available upon request, select 'No' for the first question and explain your exceptional situation in the text box.<br><br>Do the authors confirm that all data underlying the findings described in their manuscript are fully available without restriction?	
**Describe where the data may be found in full sentences. If you are copying our sample text, replace any instances of XXX with the appropriate details.**<br><br>• If the data are **held or will be held in a public repository**, include URLs, accession numbers or DOIs. If this information will only be available after acceptance, indicate this by ticking the box below. For example: *All XXX files are available from the XXX database (accession number(s) XXX, XXX.*).<br>• If the data are all contained **within the manuscript and/or Supporting Information files**, enter the following: *All relevant data are within the manuscript and its Supporting Information files.*<br>• If neither of these applies but you are able to provide **details of access elsewhere**, with or without limitations, please do so. For example:<br><br>*Data cannot be shared publicly because of [XXX]. Data are available from the XXX Institutional Data Access / Ethics Committee (contact via XXX) for researchers who meet the criteria for access to confidential data.*<br><br>*The data underlying the results presented in the study are available from (include the name of the third party*	All relevant data are within the manuscript and its Supporting Information files

and contact information or URL).<br>• This text is appropriate if the data are owned by a third party and authors do not have permission to share the data.<br><br>* typeset	
Additional data availability information:	

Manuscript Click here to access/download;Manuscript;Manuscript...docx

Prevalence and determinants of care needs among older adults in Ghana.

Kofi Awuviry-Newton[1]; Kwadwo Ofori-Dua[2]; Charles Selorm Deku[2]; Kwamina Abekah-Carter[3*]; Victoria Awortwe[4]; George Ofosu Oti[3]

[1]Priority Research Centre for Generational Health and Ageing, Faculty of Health and Medicine, University of Newcastle, Australia;

[2]Department of Sociology and Social Work, Kwame Nkrumah University of Science and Technology, Kumasi, Ghana;

[3]Department of Social Work, University of Ghana, Legon, Ghana;

[4]Department of Social Studies, Universitetet I Stavanger, Stavanger, Norway

*Corresponding Author. Email: kwamina29@gmail.com

Prevalence and determinants of care needs among older people in Ghana

Abstract

Given the increase in longevity among older people in Ghana, as well as the likely occurrence of functional disability in their later years, the understanding of the care needs of Ghanaian older people have become more critical. This cross-sectional study examined the prevalence and determinants of care needs among older people in Ghana. Data was gathered from a sample of 400 older people from the Komfo Anokye Teaching Hospital in Southern Ghana. Care needs were assessed by one question "Do you regularly need help with daily tasks because of long-term illness, disability or frailty?" Multivariate logistic regression was used to test the association between care needs and independent variables. The majority of the respondents (81%), particularly women (54%) reported a need for care in completing their daily tasks. The determinants of care needs were mainly functional disability (AOR=1.07 95%CI: 1.05-1.09, $p<0.001$) and the lack of government support (AOR=3.96 95%CI: 1.90-8.25, $p<0.001$). Functional disability and absence of government support are the major factors that need to be prioritized in addressing increased demand for care related to daily tasks among older people in Ghana.

Key Words: Care needs, WHO-ICF, older people, Ghana

Introduction

Ghana is a developing country in Africa, and the population aged 60 years and older in Ghana is growing rapidly due to decreasing birth rates and delayed mortality [1]. The number of older people in Ghana increased more than seven-fold from 213,477 (4.5%) in 1960 to 1,643,381 (6.7%) in 2010 [2]. The percentage of older people in Ghana is further expected to increase to 9.8% by 2050 [3]. Although the percentage of older people in Ghana is low compared to developed countries, it is worthy to note because the pace of ageing in this geographical territory is faster [4]. Population ageing is associated with increased service needs; therefore, it requires updating policies and programs to respond to the current and future health needs of older people in Ghana [5], particularly those who may be living with a functional disability.

Given the increase in longevity among older people in Ghana [1,3], and the likely occurrence of functional disability in older people's later years [6] including older Ghanaians, the understanding of their care needs have become more critical. Care needs can be identified as the need for assistance required in essential life activities, including activities of daily living (ADL) and instrumental activities of daily living (IADL) [7,8]. Independence in ADLs and IADLs are essential in promoting the health and social well-being of older people. However, there is a paucity of knowledge in Ghana about the prevalence and determinants of care needs among older people, obscuring policymakers, health professional and program developers' understanding of how to promote functional abilities or promote the wellbeing of this vulnerable population.

In Ghana, the decline of the traditional extended family support system for older people is a significant trend [9, 10]. Before colonisation, life in Ghana was characterised by a patriarchal social system based on kinship [10,11]. Solidarity and mutual respect were the core features of the traditional Ghanaian way of life [12,13]. The extended family system as a venerated institution, offered social and economic support to older people when they were in need of care,

especially those living with disabilities, those living alone, and those experiencing financial hardship [9,14]. A number of factors accounted for the breakdown of traditional extended family care and support for older people in Ghana. The first reason is migration, and Van der Geest [15] in his work, gave an account of how the movement of farmers to other rural areas where they could acquire vast tracts of arable land led to the separation of family members. The second factor is urbanisation, which refers to the movement to urban centres or abroad accounting for the breakdown in traditional family ties [12, 16, 17]. Due to the breakdown of traditional family support, usually, one member of the family has to become a primary caregiver for older people who may need care [10,15], and this is costly and burdensome [18]. Often, these caregivers report facing challenges such as poor health, psychological instabilities and financial difficulties due to the caregiving [18]. Consequently, the assessment of care needs of older people is essential to inform policymakers so as to formulate and implement relevant policies and programs that would aim to improve the support system of older people in Ghana.

Globally, there are a number of studies that have examined factors associated with care needs among older people. The literature has distinctively reported personal, health and environmental factors as influences of care needs among older people [19, 20, 21]. For instance, Rosso, Auchincloss, and Michael [22] reported that favourable transportation and mobility difficulties are associated with high care needs among older people. Similarly, other researchers identified disability-unfriendly physical environment as a main factor that account for high care needs among older people [19, 20]. The personal factors identified included living in the urban areas [23], being a woman [24], living closer to own children [25,26,27], being older and living alone [21], being divorced [28] and a low level of education [29]. The main health related factors reported included having difficulties in ADLs, IADLs, living with depression [20, 27, 30, 31].

In Ghana, what we do know is that few studies exist on assessment of functional difficulties among older people [32, 33, 34]. For instance, one quantitative study reported that older women experience functional difficulties compared to older men and older people aged more than 90 were more likely to report poor health status [35]. Similarly, a mixed-method Ghanaian study reported that participants, mostly those aged 60 or older, experience participation restrictions in voluntary work, including childcare due to functional difficulties. To the best of the authors' knowledge, no study have been conducted on care needs and its determinants in Ghana. Moreover, no study employed a holistic conceptual framework like the World Health Organisation International Classification on Functioning, Health, and Disability (WHO-ICF) to determine the potential determinants of care needs among older people in Ghana.

According to the World Health Organisation (WHO), the WHO-ICF refers to a conceptual framework essential for describing and understanding a person's health in terms of function and disability [36]. Function refers to all body functions, activities and participation without restrictions, and disability refers to the impairments, activity limitations and participation restrictions [36]. The WHO-ICF acknowledges the interaction between determinants of health and disability with personal and environmental factors [36,37]. This framework perceives that the interactions of these entire components help in understanding the health needs of people, including older people. Accordingly, the factors determining the care needs of older people will encompass environmental, health status, community, and network factors, as well as the intrinsic capacities of older people.

This current study explored care needs in the context of the components of WHO-ICF. This information will enhance the understanding of the holistic factors determining older people's care needs. We hypothesized that the higher the need across the components of the WHO-ICF, the more likely older people would need care.

Research Design and Methodology

Study design, setting and sample

A cross-sectional study was conducted in the Komfo Anokye Teaching hospital, Ashanti Region, Ghana. The minimum sample size required in this study was 200. The participants were older people aged 60 years or over, admitted to the above hospital due to any health problem or frailty, had stayed a minimum of one night in any ward of the hospital, and had expressed their willingness to participate in this study. Patients who were seriously ill were excluded. The study employed a consecutive sampling technique based on the hospital admission register, and 400 older people participated during the data collection period.

Recruitment of participants was completed on randomly selected days (i.e., 4 days per week), and lasted for over eight hours per day to increase the likelihood of giving every older person who was admitted at the facility the opportunity to participate. After the hospital nurses provided assistance in determining the eligibility of older people to partake in the study, the primary researcher recruited participants for the study. Data were collected during participants' hospitalisation at a day and time chosen by the participants.

Data collection

Data were collected during the months of April to August 2018. A survey questionnaire was used to collect data from all participants. A self-administered questionnaire was used to collect data from literate participants. The primary researcher read the questions to solicit responses from participants who were illiterate. The questionnaire consisted of the socio-demographic profile, information related to their hospital admission, care needs and functional ability level.

Measures

Dependent variable

Care needs

In this study, care needs were assessed by one question, "Do you regularly need help with daily tasks because of long-term illness, disability or frailty (e.g., personal care, getting around, preparing meals, etc).?" based on dichotomous responses "Yes" and "No".

Independent variables

Based on the WHO-ICF, the six domains with their respective variables are discussed below:

Personal factors

According to the WHO-ICF, personal factors refer to the intrinsic nature of individuals [37]. In this research, personal factors included age, gender, marital status, education, religion, and employment status. In this study, it was hypothesised that older people who were advanced in age, were females, lived as widows, had no religious affiliation, had a low level of education, lived in the urban area, living alone, and not working would report high care needs.

In this study, age was measured as a continuous variable. Gender was measured on a categorical nominal variable "male" or "female". Marital status was measured with "never married", "married/cohabiting", "separated/divorced", and "widowed". Marital status was categorised as "single", "separated or divorced", "married or cohabiting", and "widowed" for chi-square analysis. In terms of education, the response categories were "no education", "at most Junior High completed", "Senior High completed" and "at least College/Pre-University completed". These categories were further categorised as "no education", "at most Junior High completed", and "at Least Senior High School completed". Religion was initially measured as "none", "Christianity (including Roman-Catholics)", "Islam", and "Traditional religion". However, for the analysis, the "none" and "Traditional religion" were combined as "none", with

"Christianity" and "Islam" treated as separate categories. In terms of living arrangements, the categories were "alone", "as a couple", "as a couple and with children". Employment status was categorised as "currently working" and "currently not working".

Body function and structure

Body function and structure refers to the level of impairment or function of individuals that can influence their overall health [37]. The body function and structure was assessed by one open-ended question; "What physical impairment did the doctor/nurse diagnose you with……….?" This was also used to determine any impairment among the participants. The responses reflecting this component was mainly "visual impairment" and "injury". These variables were coded as "1" meaning "Yes" and "0" meaning "No".

Health conditions

Health condition refers to any illness or chronic condition that can affect the overall functioning of an older person [37]. In this study, health conditions were assessed by one open-ended question "What illness did the doctor/nurse diagnose you with…?" These conditions include chronic condition, infectious diseases and alcoholism. The health conditions mainly reported by participants were diabetes, stroke, ulcers, cancer, hypertension, kidney disease, asthma, heart disease, and lung diseases. Other health conditions reported were hernia, malaria, chronic alcoholism, heart failure, jaundice, atrial fibrillation, ganglia, urosepsis, haemorrhage, gastroenteritis, uremic encephalopathy, anaemia, urinary tract infection, cardiomyopathy, goitre, chronic left external capsular infarct, appendicitis, liver disease, pneumonia, intestinal lung disease, cellulitis, gangrene, hepatitis, cataract, fall injury, kidney disease, blindness, fibroid, angina, prostate enlargement, and neurological problem. Each of these variables' responses were coded as 1(presence of medical problems and 2 (not present). For the analysis,

these conditions were transformed into multi-morbidity variable coded as “no condition”, “at least one condition” and “two or more conditions”.

Activity limitations

According to the WHO-ICF, activity limitation refers to difficulties experienced by people in carrying out life activities [37]. Activity limitation was assessed by a 24 items questionnaire, consisting activity of daily living (ADLs), and instrumental activities of daily living (IADLs) [38]. Difficulties were graded using ordinal response categories (none (0), mild (1), moderate (2), severe (3), extreme (4)) in response to the question “In the last 30 days, how much difficulty did you have with ………due to health problems, injuries, mental or emotional problems?” Internal consistency of response across the 24 items in this study was assessed, and reliability was found to be high (Cronbach alpha= 0.96).

Environmental factors

According to the WHO-ICF, environmental factors refer to the physical, social and attitudinal aspects that influence individual function [37]. In this study, the social environment was assessed by two questions, (1) “How many children do you have? The response was categorised as “none”, “1-5”, and “6 or more”, and (2)”Do you receive support from neighbours or community, or government, or religious members, or non-governmental organisation? Emotional support was assessed by one question “How many times during the past week did you spend time with someone who does not live with you, that is, you went to see them, or they came to visit you, or you went out together?” based on eight points Likert Scale (0 to 7). (eg. Frequency) to 7 or more, but was categorised as “none”, “1-5 times” and “6 or more times” for analysis. Perceived social support was measured using nine questions based on three-point scales “hardly ever”, “some of the time”, and “most of the time”. For example, “Does it seem that your family and friends, people who are important to you understand you?

Participation

Participation refers to the level of engagement in social or community activities to increase functioning [37]. In this study, the participation of social or community activities was assessed by one question "About how often did you go to meetings of clubs, religious meetings or other groups that you belong to in the past week?", based on a scale from "none" to "seven or more". These responses were categorized as "none", and "at least once".

Data analysis

Descriptive statistics were used to describe the demographic information of study participants. To compare relationships between categorical variables, a bivariate analysis was utilised. Bivariate and multivariable logistic regression was performed to assess any significant relationship between variables under WHO-ICF components (independent variables) and care needs (dependent variable). Logistic regression was used to estimate crude and adjusted odds ratios and 95% confidence intervals. Any variable with a p-value of 0.2 in the bivariate association was considered for inclusion in the multivariate logistic regression analysis. A p-value of 0.05 was used to identify the determinants of care needs. Stata version 15 was used to manage the analysis.

Results

The demographic characteristics of the participants are presented in Table 1. Participants were mainly women (51%), and the average age was 72 years. Furthermore, 81% of participants reported a need for care in daily living tasks (See Table 1 for further information).

[Insert Table 1 here]

Bivariate analysis of care needs with variables across WHO-ICF component

From the bivariate analysis, marital status, religious belief, residence, living arrangement, visual impairment, injury, perceived support variables, religious group/member support, non-governmental organisation support, and several children were not significant with care needs. More than half (51%) of the participants who reported care needs lacked governmental support (see Table 2).

[Insert Table 2 here]

Determinants of care needs among older people

In the unadjusted logistic regression, age, gender, education, employment status, multi-morbidity, disability score, and participation restriction status were significant determinants of care needs among older people. Besides, speaking with someone via the phone, spending time with someone whom older people do not live with, and government support were statistically significantly associated with older people' need for care (see Table 3).

[Insert Table 3 here]

However, adjusting for all these statistically significant variables, functional disability score (AOR=1.07 95%CI: 1.05-1.09, $p<0.001$) and government support (AOR=3.96 95%CI: 1.90-8.25, $p<0.001$) were independently statistically significantly associated with the need for care among older people.

Regarding the disability score, a 1-unit increase in disability score increases the odds of older people needing care by 7%. Concerning government support, there was 3.96% more likely for those who lacked governmental support to report needing care. These were statistically significant.

Discussion

To the best of our knowledge, this is the first reported study that has selected variables based on the WHO-ICF framework to identify factors influencing care needs among older people in Ghana. Overall, the findings revealed that care needs are related to environmental factors and activity limitation domains of the WHO-ICF framework. Variables under the personal factors, such as body function and structure, participation, and health condition components of the WHO-ICF were not significantly associated with care needs in this study. Thus, it is highly recommended that interventions designed to meet the care needs of older people should be targeted at addressing health-related factors (functional disability), and environmental factors that increase the prevalence of care needs.

Furthermore, the prevalence of care needs among older people in this study, was high (81%), with a higher prevalence recorded among the older women than the older men. This finding is similar to that of Debpuur et al. [35], which revealed that older women report poor health as compared to older men. This study finding implies that majority of older people in Ghana may be in need of care, especially in completing vital daily activities. Thus, this finding is a stepping-stone to inform policymakers, social welfare program developers and health care professionals in Ghana to develop innovative interventions or approaches to meet the needs of older people. Most importantly, formulators of such interventions should take into consideration the gender variation of care needs among the aged population.

The finding that one additional increase in disability score is related to a 7% increase in odds of care needs reveals how health-related factors could deteriorate the quality of life older people in Ghana. This finding is consistent with previous studies conducted globally that functional disability increases care needs among older people [19,20]. Older people living with a functional disability may experience restrictions in participating in everyday activities they may like to do. Activities needed to improve older people's health, such as visiting friends and

attending social gathering may be impacted, and this tends to increase the need for care. Health insurance programs should be established to increase the accessibility of healthcare among older people in Ghana. There should be a promotion of national interest in the health needs of older people to enhance older people's independence and wellbeing.

In this study, it was also revealed that government support is significantly associated with older people's care needs. By implication, the absence of government support, specifically financial will mean older people will have a high need for care. This is understandable in Ghana because the traditional extended family that was providing care and support for older people in times of disability is reportedly depleting [9,10], drawing attention to the need for government to provide health, financial, emotional and material support for older people. Absence of government support may be catastrophic for older people in the future because they will be left alone to care for themselves.

In this study, personal factors, such as advanced age, lower level of education, being divorced or living alone were not statistically significant in the adjusted model. This finding contradicts findings of other multivariate logistic regression studies that revealed a significant relationship between these variables and care needs among older people [21, 28, 29]. The difference in the relationship between age and care needs may be because participants were recruited from a hospital and they were all aged 60 years or older. Therefore, their self-reported care needs may be influenced by their functional disability rather than their age.

The current study has several implications. First, the study's findings present evidence for researchers to explore the government's interest in addressing the health and social service needs of older people in Ghana. It also implies that more research needs to be conducted on functional disabilities among older people in Ghana. Qualitative studies exploring the narratives of older people concerning their functional disabilities and need for care are

essential, as it will provide empirical evidence to draw the attention of relevant stakeholders on the need to urgently intervene and enhance the welfare of older people in Ghana. This study has some limitations that need to be acknowledged. Thus, it used a small sample size, and the findings may not apply to the general population of older people in Ghana.

Moreover, this study did not model the interaction across the domains of the WHO-ICF; however, this was necessary because the reason for employing the WHO-ICF was to serve as a guide for variable selections. Furthermore, this study did not include the physical environment that may also determine the level of care needs among older people. More research to model the various components of the WHO-ICF is essential to understand its overall impact on care needs.

Conclusion

Prevalence of care needs is high among hospitalised older people in general, particularly older women in Ghana. Care needs was found to be associated with functional disability and government support. These identified factors influencing care needs revealed in this study when tackled, might help meet the care needs of older people in Ghana. These findings have drawn attention to the need for a multi-sectorial interest in the health and social care needs of older people in Ghana.

References

1. Biritwum RB, Mensah G, Yawson AE, Minicuci N. (2013). Study on global AGEing and adult health (SAGE), Wave 1: the Ghana national report. Geneva: World Health Organization; 2013

2. Ghana Statistical Service. Population and Housing Census Report. 2013. Retrieved from www.statsghana.gov.gh/docfiles/2010phc/National_Analytical_Report.pdf. Retrieved 26 March, 2017, from Ghana Statistical Service, Accra www.statsghana.gov.gh/docfiles/2010phc/National_Analytical_Report.pdf

3. United Nations, Department of Economic and Social Affairs. World population prospects, the 2017 Revision, Volume I: comprehensive tables. 2017.

4. He W, Goodkind D, Kowal, PR. An aging world: 2015: United States Census Bureau; 2016.

5. Araujo de Carvalho I, Byles J, Aquah C, Amofah G, Biritwum R, Panisset U, et al. Informing evidence-based policies for ageing and health in Ghana. Bulletin of the World Health Organization, 2015;93(1): 47-51.

6. Christensen K, Doblhammer G, Rau R, Vaupel JW. Ageing populations: the challenges ahead. The lancet. 2009;374(9696):1196-1208.

7. Beach SR, Schulz R. Family caregiver factors associated with unmet needs for care of older adults. Journal of the American Geriatrics Society. 2017;65(3):560-566.

8. Williams J, Lyons B, Rowland D. Unmet long-term care needs of elderly people in the community; A review of the literature. Home Health Care Services Quarterly. 1997;16(1-2):93-119.

9. Nukunya GK. Tradition and Change in Ghana: An Introduction to Sociology. Accra: University Press; 2003.

10. Ofori-Dua K. Extended Family Support and Elderly Care in Bamang, Ashanti Region of Ghana. (PhD): University of Ghana; 2014.

11. Ray DI. Ghana: politics, economics and society: Burns & Oates; 1986.

12. Nyanguru AC. Aging and the elderly in the family: A changing African Scenario. In Changing family systems: A global perspective. (Tapogo M., Levers, L.L. & Jacques, G., (Eds.) ed.): Botswana: Bay Publishing, Gaborone; 2008

13. UNICEF. Social protection and children: Opportunities and challenges in Ghana: Accra; 2009.

14. Kumado K, Gockel, AF. A study on social security in Ghana. Accra: Friedrich Ebert Stiftung; 2003.

15. Van der Geest S. Respect and reciprocity: Care of elderly people in rural Ghana. Cross-Cultural Gerontology. 2002;17:3-31.

16. Brown CK. Caring for the elderly: perspectives from Ghana and Japan. Cape Coast: Catholic Mission Press; 1999.

17. Oppong C. (2006). Familial roles and social transformations: Older men and women in sub-Saharan Africa. Research on Aging. 2006;28(6):654-668.

18. Sanuade OA, Boatemaa S. Caregiver profiles and determinants of caregiving burden in Ghana. Public Health. 2015;129(7):941-947.

19. Briller S, Calkins MP. Defining place-based models of care: Conceptualizing care settings as home, resort, or hospital. Alzheimer's Care Today. 2000;1(1):17-23.

20. Hoogendijk EO, Muntinga ME, van Leeuwen KM, van der Horst HE, Deeg DJ, Frijters DH, et al. Self-perceived met and unmet care needs of frail older adults in primary care. Archives of Gerontology and Geriatrics. 2014;58(1):37-42.

21. Ortman, J. M., Velkoff, V. A., & Hogan, H. (2014). An aging nation: the older population in the United States: United States Census Bureau, Economics and Statistics Administration, US ….

22. Rosso AL, Auchincloss AH, Michael YL. The urban built environment and mobility in older adults: a comprehensive review. Journal of Aging Research, 2011.

23. Agrawal G, Patel SK. Religious differentials in morbidity prevalence and health care seeking behaviours among older persons in India. International Journal of Human Rights in Healthcare. 2017.

24. West L, Cole S, Goodkind D, He W. 65+ in the United States: 2010. Special studies: Current population reports. Washington, DC: United States Census Bureau. 2014.

25 Spillman BC, Pezzin LE. Potential and active family caregivers: Changing networks and the 'sandwich generation'. The Milbank Quarterly. 2000;78(3):347-374.

26. Wee SL, Li Y, Sun Y, Chua YX. Determinants of Long- Term Care Needs of Community-Dwelling Older People in Singapore. Journal of the American Geriatrics Society. 2014;62(12):2453-2454.

27. Wee SL., Liu C, Goh SN, Chong WF, Aravindhan A, Chan A. Determinants of use of community- based long- term care services. Journal of the American Geriatrics Society. 2014;62(9):1801-1803.

28. Brown SL, Lin, IF. The gray divorce revolution: Rising divorce among middle-aged and older adults, 1990–2010. The Journals of Gerontology: Series B. 2012;67(6):731-741.

29. Schulz R, Eden J. Families caring for an aging America: National Academies Press Washington, DC; 2016.

30. Freedman VA, Spillman BC. Disability and care needs among older Americans. The Milbank Quarterly. 2014:92(3):509-541.

31. Taylor BD, Tripodes S. The effects of driving cessation on the elderly with dementia and their caregivers. Accident Analysis & Prevention. (2001);33(4):519-528.

32. Amosun S, Nyante G, Wiredu E. Perceived and experienced restrictions in participation and autonomy among adult survivors of stroke in Ghana. African Health Sciences. 2013;13(1):24-31.

33. Biritwum R, Minicuci N, Yawson A, Theou O, Mensah G, Naidoo N, et al. Prevalence of and factors associated with frailty and disability in older adults from China, Ghana, India, Mexico, Russia and South Africa. Maturitas. 2016;91:8-18.

34. Peltzer K, Hewlett S, Yawson A, Moynihan P, Preet R., Wu, F, et al. Prevalence of loss of all teeth (edentulism) and associated factors in older adults in China, Ghana, India, Mexico, Russia and South Africa. International Journal of Environmental Research and Public Health. 2014;11(11):11308-11324.

35. Debpuur, C., Welaga, P., Wak, G., & Hodgson, A. (2010). Self-reported health and functional limitations among older people in the Kassena-Nankana District, Ghana. Global Health Action, 3, 54-63. doi:10.3402/gha.v3i0.2151

36. World Health Organization. International Classification of Functioning, Disability and Health: ICF: World Health Organization; 2001.

37. World Health Organization. Towards a common language for functioning, disability and health: ICF. 2002. 2005. Available at: www3. who. int/icf/beginners/bg. pdf. Accessed August, 5.

38. Kowal P, Chatterji S, Naidoo N, Biritwum R, Fan W, Lopez Ridaura R, et al. Data resource profile: the World Health Organization Study on global AGEing and adult health (SAGE). International Journal of Epidemiology, 2012;41(6):1639-1649.

Table 1. Demographic characteristics of older people, and care needs

Demographic Characteristics (N=400)	N (%)
**Age** *(mean, SD)*	71.3±8.42
**Sex**	
Male	196 (49.0
Female	204 (51.0)
**Marital status**	
Single/separated/divorced	58 (14.5)
Currently married/cohabiting	212 (53.0)
Widowed	130 (32.5)
**Education**	
No education	128 (32.0)
At maximum junior high completed	209 (52.3)
At least senior high completed	63 (15.8)
**Religion**	
None	27 (6.75)
**Christianity**	331 (82.8)
Islam	42 (10.0)
**Residence**	
Rural	227 (56.8)
Urban	173 (43.3)
**Living arrangement**	
Alone	50 (12.5)

With couple	100 (25.0)
With couple and children	250 (62.5)
**Employment status**	
Currently working	156 (39.0)
Currently not working	244 (61.0)
**Do you need care?**	
Yes	322 (80.5)
No	78 (19.5)

Table 2: Bivariate analysis of care needs across WHO-ICF components

Demographic Characteristics (N=400)	Total N (%)	care need		p-value
**PERSONAL FACTORS**		Yes N (%)	No N (%)	
**Age *(mean, SD)***	71.3±8.42	71.8±8.45	68.9±7.93	0.006
**Sex**				0.027
Male	196 (49.0)	149 (46.3)	47 (60.3)	
Female	204 (51.0)	173 (53.7)	31 (39.7)	
**Marital status**				0.623
Single/separated/divorced	58 (14.5)	45 (14.0)	13 (16.7)	
Currently married/cohabiting	212 (53.0)	169 (52.5)	43 (55.1)	
Widowed	130 (32.5)	108 (33.5)	22 (28.2)	

**Education**				0.006
No education	128 (32.0)	110 (34.2)	18 (23.1)	
At least junior high completed	209 (52.3)	170 (52.8)	39 (50.0)	
At least senior high completed	63 (15.8)	42 (13.0)	21 (26.9)	
**Religion**				0.933
None	27 (6.75)	21 (6.52)	6 (7.69)	
**Christianity**	331 (82.7)	267 (82.9)	64 (82.1)	
Islam	42 (10.5)	34 (10.6)	8 (10.3)	
**Residence**				0.659
Rural	227 (56.8)	181 (56.2)	46 (59.0)	
Urban	173 (43.3)	141 (43.8)	32 (41.0)	
**Living arrangement**				0.842
Alone	50 (12.5)	41 (12.7)	9 (11.5)	
With couple	100 (25.0)	82 (25.5)	18 (23.1)	
With couple and children	250 (62.5)	199 (61.8)	51 (65.4)	
**Employment status**				0.006
Currently working	156 (39.0)	115 (35.7)	41 (52.6)	
Currently not working	244 (61.0)	207 (64.3)	37 (47.4)	
**BODY FUNCTION AND STRUCTURE**				
**Visual impairment**				0.268
Yes	5 (1.25)	5 (1.55)	0 (0.00)	
No	395 (98.8)	317 (98.5)	78 (100)	
**Injury**				0.212

Yes	265 (66.3)	218 (67.7)	47 (60.3)	
No	135 (33.8)	104 (32.3)	31 (39.7)	
**CHRONIC HEALTH CONDITION**				
**Multi-morbidity**				0.138
At most 1 condition	308 (77.0)	243 (75.5)	65 (83.3)	
Any 2 or more conditions	92 (23.0)	79 (24.5)	13 (16.7)	
**ACTIVITY LIMITATION**				
Disability score (mean, SD)	54.6±21.2	60.1±16.9	31.9±21.9	<0.001
**ENVIRONMENTAL FACTORS**				
Perceived Support				
**Family and friend understand you**				0.809
Hardly ever	74 (19.0)	62 (19.6)	12 (16.4)	
Some of the time	162 (41.7)	130 (41.1)	32 (43.8)	
Most of the time	153 (39.3)	124 (39.2)	29 (39.7)	
**Feel useful to family and friends**				0.742
Hardly ever	83 (20.8)	68 (21.1)	15 (19.2)	
Some of the time	173 (43.3)	141 (43.8)	32 (41.0)	
Most of the time	144 (36.0)	113 (35.1)	31 (39.7)	
**Awareness of matters concerning family and friends**				0.381
Hardly ever	179 (44.8)	142 (44.1)	37 (47.4)	
Some of the time	112 (28.0)	95 (29.5)	17 (21.8)	

Most of the time	109 (27.3)	85 (26.4)	24 (30.8)	
**Share deepest problems with some family and friends**				0.387
Hardly ever	83 (20.8)	67 (20.8)	16 (20.5)	
Some of the time	173 (43)	144 (44.7)	29 (37.2)	
Most of the time	144 (36.0)	11 (34.5)	33 (42.1)	
Emotional support				
**Often time you spoke with someone via telephone (past week)**				<0.001
None	173 (43.3)	154 (47.8)	19 (24.4)	
1-5 times	161 (40.3)	123 (38.2)	38 (48.7)	
6 or more times	66 (16.5)	45 (14.0)	21 (26.9)	
**Spent time with someone who does not live with you (past week)**				0.034
None	54 (13.5)	47 (14.6)	7 (8.97)	
1-5 times	196 (49.0)	164 (50.9)	32 (41.0)	
6 or more times	150 (37.5)	111 (34.5)	39 (50.0)	
**Neighbours/community support**				0.110
Yes	222 (55.5)	185 (57.5)	37 (47.4)	
No	178 (44.5)	137 (42.6)	41 (52.6)	
**Government support**				<0.001
Yes	174 (43.5)	158 (49.1)	16 (20.5)	
No	226 (56.5)	164 (50.9)	62 (79.5)	

**Religious group/members support**				0.216
Yes	235 (58.8)	194 (60.3)	41 (52.6)	
No	165 (41.3)	128 (39.8)	37 (47.4)	
**Non-government organisation support**				0.443
Yes	10 (2.50)	9 (2.80)	1 (1.28)	
No	390 (97.5)	313 (97.2)	77 (98.7)	
**Number of children**				0.554
At most one child	40 (10.0)	31 (9.63)	9 (11.5)	
2-4	128 (32.0)	100 (31.1)	28 (35.9)	
5 or more	232 (58.0)	191 (59.3)	41 (52.6)	
**PARTICIPATION**				
**Often times you attend meetings (past week)**				0.006
None	313 (78.3)	261 (81.1)	52 (66.7)	
At least once	87 (21.8)	61 (18.9)	6 (33.3)	

Table 3. Determinants of care needs among older people in Ghana

Variables based on ICF component	Unadjusted OR; 95% CI	Adjusted OR, 95% CI
**PERSONAL FACTORS**		
**Age**	1.05 (1.01, 1.08)**	1.01 (0.97, 1.06)
**Gender**		
Female (vs male)	1.76 (1.06, 2.91)*	0.99 (0.48, 2.10)
**Education**		
No education (vs At least senior high completed)	3.06 (1.48, 6.30)**	1.83 (0.66, 5.04)
at most junior high completed (vs At least senior high completed)	2.18 (1.16, 4.09)*	1.43 (0.60, 3.37)
**Employment status**		
Currently not working (vs Currently working))	2.00 (1.21, 3.29)**	1.61 (0.80, 3.22)
**HEALTH CONDITION**		
Multi-morbidity		
Any 2 or more conditions (vs at most one condition)	1.63 (0.85, 3.11)	
**ACTIVITY LIMITATION**		

Disability score	1.07 (1.05, 1.08)***	1.07 (1.05, 1.09)***
**PARTICIPATION RESTRICTION**		
Often times you attend meetings (past week)		
None(vs at least once)	2.14 (1.24, 3.70)**	0.76 (0.32, 1.79)
**ENVIRONMENTAL FACTORS**		
Emotional support		
**Spent time with someone who does not live with you (past week)**		
None (vs6 or more times)	2.36 (0.99, 5.65)	1.84 (0.62, 5.44)
1-5 times (vs 6 or more times)	1.80 (1.06, 3.05)*	1.68 (0.83, 3.40)
**Often time you spoke with someone via telephone (past week)**		
None(vs 6 or more times)	3.78 (1.87, 7.65)***	1.78 (0.70, 4.50)
1-5 times (vs 6 or more times)	1.51 (0.80, 2.85)	2.33 (0.99, 5.46)
**Neighbours/community support**		

No (vs Yes)	0.67 (0.41, 1.10)	
**Government support**		
No (vs Yes)	3.73 (2.07, 6.74)***	3.96 (1.90, 8.25)***

Significant at *p-value < 0.05; **p-value < 0.01, ***p-value<0.001
