# Supplementary material for: Prevalence and determinants of care needs among older people in Ghana
Source: PLoS One. 2022 Feb 15;17(2):e0263965. doi: 10.1371/journal.pone.0263965 (PMC8846497; doi:10.1371/journal.pone.0263965)
Supplement: Attachment — Submitted filename: Comments plos one manuscript.docx [file pone.0263965.s003.docx]

Review of plos one manuscript

introduction

1. What is the difference between instrumental ADL and ADL. Readers need to understand these terms in the introduction.
2. There is a lot mentioned about functional disability among the aged in introduction. If this is the main factor explored, then it must be mentioned in title and abstract in like manner. See highlighted portions. I see authors used a framework WHO-ICF in conducting the study. If there were specific factors such as functional disability, ADL etc in this framework that were explored, this should be made clearer in the introduction. Probably this work was about exploring those factors within the WHO-ICF framework.
3. In paragraph 3, authors stated that there is paucity of knowledge about……..this should come at the end of introduction, and then this can set the tone for the objectives of this study.
4. In paragraph 4, the authors bring in history of how Ghanaians lived in the past. This does seem to fit in here. I believe the authors want to make the point about the breakdown of family support systems for older Ghanaians in contemporary Ghana. This point can be made in a concise manner.
5. Again I do not see the point about care givers reporting poor health and psychological instability etc………..in paragraph 4. May be authors should concentrate on older people themselves and what the evidence is, about their challenges.
6. In paragraph 5, the authors state ‘globally, there are a number of studies…..’ the introduction has to be rearranged. This global piece was brought in after issues in Ghana were earlier introduced. Typically, the global aspects will be introduced then narrowed down to Africa and Ghana. This will make reading of manuscript better.
7. Again authors bring in WHO-ICF framework closer to the end of introduction. This does not quite fit in here. This should be bought up earlier and discussed in a way that shows the reasons for its usage.

Methods

1. The authors state that the minimum sample size required in this study was 200. Readers are unable to tell how this was arrived at. Which sample calculation method was used? The sampling techniques used are unclear. In one aspect, authors state consecutive sampling using hospital register and later states random selection of patients based on days they visited the hospital. This part should be clarified in methods.
2. Data collection. It will help if authors can add questionnaire as an appendix.

Plagiarism check.

This work has a 52% similarity with other works (checked with ithenticate). Authors should kindly modify to reduce the percentage of similarity with other published works.
