# Supplementary material for: Prevalence and determinants of care needs among older people in Ghana
Source: PLoS One. 2022 Feb 15;17(2):e0263965. doi: 10.1371/journal.pone.0263965 (PMC8846497; doi:10.1371/journal.pone.0263965)
Supplement: Attachment — Submitted filename: Response to Reviewers.docx [file pone.0263965.s005.docx]

Greetings to the Editor,

We do appreciate the opportunity we have been given to address reviewers’ comments to improve the manuscript. We confirm that we have addressed all the comments to the best of our ability.

Reviewer #1:

Review of Plos one manuscript
introduction
**1. What is the difference between instrumental ADL and ADL? Readers need to understand these terms in the introduction.**

**Response**

*Authors have clarified the differences between ADL and IADLs. The sentence now reads as: “Independence in ADLs (defined as basic self-care, tasks such as bathing) and IADLs (secondary task to care of self and home household responsibilities) are essential to promote the health and social wellbeing of older people.”*

**2. There is a lot mentioned about functional disability among the aged in introduction. If this is the main factor explored, then it must be mentioned in title and abstract in like manner. See highlighted portions. I see authors used a framework WHO-ICF in conducting the study. If there were specific factors such as functional disability, ADL etc in this framework that were explored, this should be made clearer in the introduction. Probably this work was about exploring those factors within the WHO-ICF framework.**

*In this study, the authors alluded to functional disability as helping to understand the care needs of older adults and supported this with relevant literature from the western countries. It was then studied as an independent variable that was potentially associating with care needs.* The *WHO-ICF framework was used in the categorisation of the independent variables. The According to the framework, the functional disability variable falls under the “activity limitation” domain.*

**3. In paragraph 3, authors stated that there is paucity of knowledge about……..this should come at the end of introduction, and then this can set the tone for the objectives of this study.**

*This section has been removed and sent to the conclusion of the introduction.*

**4. In paragraph 4, the authors bring in history of how Ghanaians lived in the past. This does seem to fit in here. I believe the authors want to make the point about the breakdown of family support systems for older Ghanaians in contemporary Ghana. This point can be made in a concise manner.**

*Thanks for this comment. Authors have deleted those fine details that are not relevant.*

**5. Again I do not see the point about care givers reporting poor health and psychological instability etc………..in paragraph 4. May be authors should concentrate on older people themselves and what the evidence is, about their challenges.**

*We decided to leave the caregivers information because that offer some understanding on the current state of care needs of older adults. For instance, will caregivers avail themselves to care for older adults? Are caregivers going through circumstance that will be challenging for them to care for their ageing care recipients?*

**6. In paragraph 5, the authors state ‘globally, there are a number of studies…..’ the introduction has to be rearranged. This global piece was brought in after issues in Ghana were earlier introduced. Typically, the global aspects will be introduced then narrowed down to Africa and Ghana. This will make reading of manuscript better.**

*Thanks for this comment. We have updated the entire introduction.*

**7. Again authors bring in WHO-ICF framework closer to the end of introduction. This does not quite fit in here. This should be bought up earlier and discussed in a way that shows the reasons for its usage.**

*Authors have separated this under analytical framework as the aim was to help in the categorisation of the variables under all components.*

Methods
**1. The authors state that the minimum sample size required in this study was 200. Readers are unable to tell how this was arrived at. Which sample calculation method was used? The sampling techniques used are unclear. In one aspect, authors state consecutive sampling using hospital register and later states random selection of patients based on days they visited the hospital. This part should be clarified in methods.**

*Thank you for your insight. Please we have addressed these comments. We have specified how the sample size was calculated. It now reads “The minimum sample size required in this study was 200 at a confidence level of 95% using Epi Info software (version 7.2.3). However, we increased this to 400 to compensate for any loss of participants.”*

*Moreover, we used two sampling techniques in this study. First, we used random sampling to select the days for data collection and after that consecutive sampling technique was used to select the participants based on the days they visited the hospital. We have clarified this sentence in the methods*.

**2. Data collection. It will help if authors can add questionnaire as an appendix.
Plagiarism check.**

*Thanks much. We have added the specific questions we used for this paper.*

**3. This work has a 52% similarity with other works (checked with ithenticate). Authors should kindly modify to reduce the percentage of similarity with other published works.**

*Thanks for this comment. Authors have taken drastic measure to reduce the similarity percentage. Thanks*

**4. Ethical considerations: I am unable to find statement on ethics in this work.**

*I have included a separate section on ethical consideration.*

Reviewer #2:

i. **In the abstract, kindly indicate the research gap calling for the study.**

*Thanks for this comment. We have provided the research gap in the abstracts. It now reads as “Given the longevity noticed among older people in Ghana, and the potential occurrence of functional disability in later years of lives, it has become essential to understand their care needs.”*

**ii. Under the data analysis section, the authors indicate that “ any variable with a p-value of 0.2 in the bi-variate association was considered for inclusion in the multivariate analysis” Is there any basis or theoretical justification for this? This should be discussed in the manuscript.**

*In public health discipline selecting a p-value of 0.2 is a common practice (Awuviry-Newton, Tavener, Wales, & Byles, 2020).*

**iii. In the methods, I did not see how the authors did measure validity and reliability of the data collection tool/ tool.**

*International consistency for the group variable (functional disability) was assessed and it was found reliable and valid. Please see under “Activity limitations”.*

**iv. In the discussion, this is how the authors should discuss the results on the prevalence of care needs.
a. The authors should tell readers whether the prevalence of care needs reported in this study is higher than what has been reported elsewhere and then assign reasons for the differences. The authors should tell readers whether the prevalence of care needs reported in this study is the same as what has been reported elsewhere.**

*Thanks for this suggestion. Authors have addressed these comments in the discussion. We could not get results on prevalence of care needs per say. However, we had some evidence to extend the discussion.*

**b. The authors should tell readers whether the prevalence of care needs reported in this study is lower than what has been reported elsewhere and then assign reasons for the differences.**

*We have addressed this comment.*

**v. A paragraph of the discussion should be devoted for the specific contribution of the study to literature.**

*This suggestion has been addressed.*

**vi. The authors should also discuss the strengths of the study.**

*Thank you. We have addressed this comment.*

**vii. The authors should further discuss how the limitations of the study did not affect the findings of the study.**

*Thanks. We have addressed this comment.*

**viii. In the conclusion section, the authors did indicate that the prevalence of care needs is high among hospitalized older people. Is there any theoretical or conceptual basis for determining the prevalence at which care needs are high?**

*Thank you for this comment. This was the overall conclusion, which is thoroughly discussed in the discussion session. Thanks*
